# 

**Published:** 1891-07

**Authors:** 


					﻿Vol. XI.
JULY, 1891.
NO. 7J
THE
HOMOEOPATHIC pHY^lClAJI,
A Monthly Journal of Homoeopathic Materia Medica
and Clinical Medicine.
“He is a freeman whom truth makes free, and all are slaves beside."
“ Seek the Truth : come whence it may, cost what it will."
EDITED AND PUBLISHED BY
WALTER M. JAMES, M. 3D.,
AND
George H. Clark, m. d.
PHILADELPHIA :
No. lies SPRUCE STREET,
LONDON
ALFRED HEATH <fc CO., Sole Agents for Great Britain,
114 Ebury Street, Eaton Square.
JIS* Yearly Subscription, $2.50, invariably in advance. Single numbers > 25 cts.
Entered at the Philadelphia Poet-Office ai Second- Claaa Mail Matter.
				

